# A Companion to the Classification of Mental Disorders

**DOI:** 10.1192/pb.bp.114.046763

**Published:** 2014-10

**Authors:** Deborah Cooper

**Figure:**
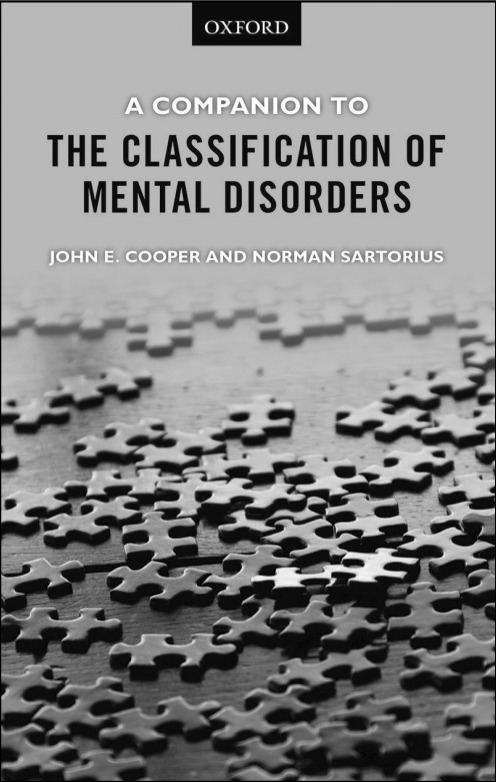


This book is timely, coinciding with the publication of DSM-5 and the pending publication of ICD-11. Perhaps wisely, it steers clear of debating what should (or should not) be included within these classifications. Instead, this slim guide aims to further our understanding of how international classification systems have developed over time, and how they can be best used by working psychiatrists.

The book is divided into a large number of small chapters and includes several useful appendices. This helps accessibility, allowing the reader to dip in and out of sections of interest, though perhaps the resulting reading experience is a little broken in terms of narrative style.

The authors begin by highlighting the pitfalls that occur in the absence of an internationally accepted classification system. This serves to reinforce the importance and relevance of classification systems today. The book then continues with several chapters that explore the history of classification. Topics covered include the development of rating scales, progress made through international epidemiological projects, and the development of the classification systems of ICD and DSM. The second half of the book looks at the construction of current classification systems. Challenges occurring in this process are highlighted, such as the existence of a large number of sub-committees, each keen for ‘their’ disorder to be fully accommodated. The issue of individual personality differences within committees is discussed, and tolerance of different approaches is suggested as essential. These chapters go some way to exploring current controversy in classification.

The authors’ conclusions are centred around the best use of classification systems, suggesting that psychiatrists need a strong knowledge of their patient and understanding of the categories of mental disorder within a classification system. The authors also give a nod to the future, predicting that further revisions of classification will occur as scientific understanding of mental disorder improves, that more ‘super-specialist’ classifications will emerge and that new disorders will be described as society changes.

This book provides context to current classification systems, acknowledges the limitations of each and encourages the reader to think more deeply about classification.

